# Early treatment with intravenous immunoglobulins in patients with critical illness polyneuropathy: a randomized controlled, double-blinded study

**DOI:** 10.1186/cc10914

**Published:** 2012-03-20

**Authors:** R Brunner, W Rinner, R Kitzberger, T Sycha, J Warszawska, U Holzinger, C Madl

**Affiliations:** 1 Medical University of Vienna, Austria

## Introduction

Critical illness polyneuropathy (CIP) is a severe complication of critical illness. The clinical features of CIP are muscle weakness and atrophy causing delayed weaning and prolongation of the mobilization phase. Although the exact etiopathogenesis has not yet been fully elucidated, sepsis, systemic inflammatory response syndrome, and multiple organ failure seem to play an important role. CIP is diagnosed by signs of denervation in electromyography. Although there is no causal treatment for CIP, retrospective data suggest that early IgM-enriched intravenous immunoglobulin (IVIG) application may prevent or mitigate CIP. Therefore we aimed to investigate the use of IVIG in the early treatment of CIP in critically ill patients in a prospective, randomized, double-blind and placebo-controlled setting.

## Methods

In this prospective, randomized, double-blind and placebo-controlled trial critically ill patients with clinical evidence for incipient CIP, a diagnosis of SIRS/sepsis and failure of at least two organ systems were randomized to be treated either with IgM-enriched IVIG or with human albumin 1% as placebo over a period of 3 days. The primary objective was to demonstrate that administration of IVIG prevents and/or mitigates CIP in critically ill patients, measured by electrophysiological stimulation of the median, ulnar and tibial nerves on days 0, 4, 7 and 14. Electrophysiological measures were graded according to compound muscle action amplitude size (CIP score) of the respective nerve. Secondary objectives were mortality from any cause within a 28-day period and lengths of ICU stay.

## Results

Thirty-eight critically ill patients were included and randomized to either receiving IgM-enriched IVIG (*n *= 19) or placebo (*n *= 19). Baseline characteristics including CIP score on day 0 were similar between the two groups. CIP could not be improved significantly by IVIG treatment for three consecutive days, represented by similar CIP scores of all three measured nerves on days 4, 7 and 14 in the IVIG and the placebo group. Mean CIP score levels of all three nerves significantly increased from baseline to day 4 in both groups.

## Conclusion

Results suggest that early treatment with IVIG neither significantly improves CIP nor influences the length of stay or mortality in critically ill patients. Consistent with the literature, CIP deteriorated during the course of disease in critically ill patients with a diagnosis of SIRS/sepsis and failure of two organ systems.

